# Holistic exploration of reading comprehension skills, technology and socioeconomic factors in Spanish teenagers

**DOI:** 10.1016/j.heliyon.2024.e32637

**Published:** 2024-06-06

**Authors:** Juan Ramón Rico-Juan, Beatriz Peña-Acuña, Oscar Navarro-Martinez

**Affiliations:** a Department of Software and Computing Systems, University of Alicante. Ctra. Sant Vicent del Raspeig s/n, 03690, San Vicente, Alicante, Spain; b Department of Philology, University of Huelva, Avenida de las Fuerzas s/n, 21071, Huelva, Spain; c Department of Pedagogy, University of Castilla-La Mancha, Spain

**Keywords:** Information and communication technologies, Socio-economic factors, Reading literacy, Explanatory AI

## Abstract

The intricate relationship between teenagers' literacy and technology underscores the need for a comprehensive understanding, particularly in the Spanish context. This study employs explainable artificial intelligence (AI) to delve into this complex interplay, focusing on the pivotal role of reading comprehension skills in the personal and career development of Spanish teenagers. With a sample of 22,400 15-year-olds from the PISA dataset, we investigate the impact of socioeconomic factors, technology habits, parental education, residential location, and school type on reading comprehension skills. Utilizing machine learning techniques, our analysis reveals a nuanced connection between autonomy, technological proficiency, and academic performance. Notably, family oversight of technology use emerges as a crucial factor in managing the impact of digital technology and the Internet on reading comprehension skills. The study emphasizes the necessity for a balanced and supervised introduction to technology from an early age. Contrary to current trends, our findings indicate that online gaming may not contribute positively to reading comprehension skills, while moderate daily Internet use (1–4 h) proves beneficial. Furthermore, the study underscores the ongoing nature of acquiring reading comprehension and technological skills, emphasizing the need for continuous attention and guidance from childhood. Parental education levels are identified as partial predictors of children's performance, emphasizing the importance of a holistic educational approach that considers autonomy and technological literacy. This study advocates for addressing socio-economic and gender inequalities in education and highlights the crucial role of cooperation between schools and families, particularly those with lower educational levels.

## Highlights

• Online gaming is negatively related to reading comprehension skills as exposure time increases.
• Moderate daily Internet use (1–4 h) is beneficial for reading comprehension skills, while extreme use is detrimental.
• Educated parents significantly improve teenagers' reading comprehension skills.
• Increasing teenagers' autonomy and technological literacy promotes reading comprehension skills.
• Late exposure to digital environments can negatively affect reading comprehension skills.

## 1 Introduction

Currently, the relationship between teenager literacy and technology, as a foundational skill for cognitive development and academic achievement, is an issue of particular relevance and complexity [1,2]. The use of digital devices plays a key role in the distance learning of young people, so it will be essential to address this interaction [3]. The aim is to approach this situation from a multidisciplinary perspective, using explicable artificial intelligence as an ideal tool to understand the most relevant aspects of this relationship. Rigorous research and in-depth reflection will be carried out to unravel the potential of digital technology in the possibilities of strengthening the reading comprehension concerning cognitive skills of Spanish teenagers.

Reading comprehension is an essential component programmed into the education of students around the world, and its importance extends beyond academia to have a significant impact on young people's lives, both in terms of personal development, career prospects [4,5], civic participation, and effective communication. In addition, good reading comprehension facilitates cognitive skills such as problem solving, informed decision making, and the development of critical thinking skills [6]. Therefore, facilitating the development of this skill achieves the United Nations Sustainable Development Goals (SDGs), as Quality Education (04), Gender Equality (05) or Reduced inequalities (10), by providing equal educational opportunities for all [7].

While technology can be a valuable tool for learning, its excessive and unregulated use may negatively affect learning. Therefore, awareness and balance are crucial to ensure that young people optimally leverage technology for educational success. The question, however, is whether the use of digital technology and the Internet improves or harms young people's reading comprehension skills [8]. In this aspect, the control of the family environment is considered essential in order to manage this contact with digital devices from a very early age [9]. There are different arguments for acting in very different ways, so we have focused on the case of Spain because we know its socioeconomic and cultural context better. In the field of education, we understand that an inclusive model is more beneficial, promoting fairer societies and sustainable development [10]. An in-depth study with a large sample allows us to draw general conclusions that serve as a reference for educational legislation.

We have based this research on a publicly accessible database from a previously conducted European survey called *Programme for International Student Assessment* (henceforth PISA) 2018. The survey agents provide a general descriptive report of the results of European students by country based on statistical analysis. However, given the abundance of variables collected and publicly offered as microdata, there is no inferential depth in all aspects of the interrelationship between variables. This allows studies like this one, where certain variables are strategically selected, what represents a gap, and statistically interrelated to derive inferential data. Some variables were considered, such as the age at which participants first used a digital device, the time spent on networks outside school on weekdays or weekends, their proficiency in Reading comprehension, their perceived competence in Information and Communications Technology (ICT) and autonomy, the use of social networks or online games, and the participants’ perception of utility provided by the use of social networks [11].

Despite the efforts of previous research to investigate reading comprehension regarding some of these cognitive variables: literal, inferential, and evaluative [[12], [13], [14], [15]], reading comprehension needs further exploration. We have found studies addressing some variables included in this investigation, but they do not establish connections with sociodemographic aspects or the topic of ICT usage such as Rodelo and Lizárraga [16], or based on the PISA report [[17], [18], [19]]. Furthermore, no holistic research has been done on the intricate interrelation of these selection of variables with AI explainability technique.

This research extends the topic of reading comprehension performance and new technologies [20], initially discussed in Navarro-Martínez & Peña-Acuña [11] with a linear and direct approximation between the variables. Our study also considers new variables pertaining to other dimensions not previously examined, such as family environment, parents' level of education, gender considerations, geographic location or type of school, and employs more advanced techniques that allow for a deeper exploration and significant new findings.

This study holds significance as it reveals that the reading comprehension of Spanish teenagers, concerning three cognitive skills, is impacted by multiple dimensions: home, technological, skill perception, geographic, scholar and genders considerations. Therefore, this study contributes to a deeper understanding of this phenomenon by encompassing the interrelationship of more factors and prioritizing their influence on reading comprehension.

This study diverges in findings concerning reading comprehension and the variables of Playing Online Games and Gender from Borgonovi and Montt's [17] inquiry. While those authors examine PISA (2012) microdata, this study updates results based on a subsequent report, PISA (2018). Moreover, this investigation diverges from Ref. [19] study on reading comprehension and the autonomy variable based in PISA (2018). Unlike their focus on autonomy's interrelation with other factors, such as ICT literacy and various reading formats.

In addition, the use of machine learning (ML) algorithms together with explainability techniques (ML-XAI) [21] offers a number of advantages for data analysis. First, they can model a wide variety of relationships between variables, whether linear or nonlinear, and they can learn complex patterns without having to know the nature of the distributions that make up the data. Second, by providing explanations for their predictions, they offer greater transparency and confidence in the results, which can lead to a deeper understanding of the underlying phenomena.

In this article, we aim to present a detailed analysis of factors that may influence the academic reading comprehension of Spanish 15-year-olds. Although several studies have investigated some of these factors, none of them has studied them as a whole or used ML techniques with explanatory power to understand their relationships in more detail. Understanding how they are related can help us understand which habits or circumstances are desirable to encourage or discourage, depending on whether they are positive or negative. Therefore, the current study aims to answer the following research questions:

RQ1 What is the relevance of socioeconomic factors, habitus and perceived self-monitoring of technology use, parental education level, home location, and school type involved in this study in explaining reading comprehension skills in Spanish teenagers?

RQ2 What is the relationship between the values of the most important factors with respect to reading comprehension skills, and what are the advisable recommendations?

## 2 Literature review

### 2.1 Access to the use of information and communication technologies (ICT)

Technology plays a fundamental role in modern society, enhancing communication, driving the economy, and transforming education, enabling innovative and accessible learning in an increasingly digitized world [22].

In the digital age, the use of these devices has become an integral part of students' lives. The relationship between this use and academic performance is a topic of interest in current educational research. According to Anderson and Dron [23], mobile devices offer educational opportunities, but their impact on academic performance varies depending on how they are integrated into the learning process. Studies can be found in which technology improves performance in general [24] or in very specific areas in particular [25].

Research in the field of technology has mainly focused on the analysis of negative psychological aspects related to health [26]. For a comprehensive understanding of the overall educational situation, it is important not to overlook these negative aspects. Nevertheless, there is an optimistic outlook on the beneficial integration of smartphones through the micro learning paradigm [27,28].

In addition, Busch and McCarthy [29] conducted research on problematic use of digital devices. Their findings suggest that young, female, and highly educated individuals are more likely to experience problems with these devices, which mainly affect emotional health. Another related empirical study was conducted by Rodelo and Lizárraga [16] at the University of Sinaloa, Mexico. This study examined the correlation between students' academic performance and their use of social networks and the Internet as a tool to support their studies. The results showed that those who spent more time on social networks for educational purposes received higher grades. De Gagne et al. [30] investigated the positive effects of microlearning, including its relationship with social media.

Yeongmin's [31] study examined how young people's use of the Internet for communication influences their adjustment to school. The findings revealed a positive correlation between early Internet familiarity and initial adjustment to school environment. Furthermore, Lareki et al.′s [32] research, focusing on young people's emerging subjectivities and digital consumption patterns, found that over a third of participants reported no impact of technology on their performance in work and academic settings. Conversely, Limniou [33] highlighted that integrating digital devices into active learning can enhance participation and academic outcomes, while Schulz's [34] research underscored the potential of mobile applications to improve academic performance.

Shifting to the theme of autonomy, García et al. [25] proposed strategies to foster learner autonomy. Seguel [35], in contrast, investigated contexts where autonomy was discouraged, concluding that the training lacked adequate support to promote autonomy and that this affected performance. Other researchers, including Carmona [36] and Józsa et al. [37], delved into the relationship between performance and academic success. Notably, the use of technology emerged as a motivational factor for students and a catalyst for effective learning, as emphasized by Navarro et al. [38].

### 2.2 Reading comprehension skills

The PISA report evaluates various languages-related reading comprehension skills [39]. These include the ability to access and retrieve information, integrate and elaborate on ideas, and critically evaluate reliability while engaging in self-reflection for learning purposes. The assessment goes beyond mere word decoding, focusing on a deeper understanding of text by measuring student's proficiency in making inferences, identifying main and supporting ideas, and connecting information across texts. Hence, the concept of *Reading Comprehension* in this case is defined in relation to the cognitive skills that are included [40]. We will also proceed to use this concept in this meaning in this text.

Previous studies have been conducted on reading comprehension skills in various educational levels. In Landi's [12] findings, subsequent regression analyses revealed that high level skills (e.g. vocabulary and print exposure) significantly outperformed low-level skills (e.g. decoding and spelling) in adult readers. Kang et al. [13] found a strong correlation between decoding skills and reading fluency with reading comprehension, highlighting their significant roles in written text. The participants in the study were high school students, between 14 and 18 years old.

Huh [14] investigates the English reading comprehension of Korean middle school students, between 11 and 14 years old, as a second language, examining its relation with three cognitive skills: literal, inferential, and evaluative. The study correlates the level of English proficiency more strongly with English reading comprehension than with Korean, as first language (L1). In a subsequent study, Huh [15] concludes that higher level comprehension skills (literal and inferential) emerged as stronger predictors of the variance of reading comprehension compared to their lower-level comprehension skills. This test was conducted with middle school as well.

Zhao et al. [41] expose that in first grade, between 6 and 7 years old, comprehension monitoring mediated between vocabulary and syntactic knowledge, influencing reading comprehension in Chinese. In the third grade, between 8 and 9 years old, only first grade syntactic knowledge significantly predicted reading comprehension, suggesting developmental changes. Zhao et al. [42] concludes that language proficiency, higher-order cognitive abilities and word reading explained 72 % of comprehension skills in Chinese.

There is a recent study of the PISA report that has a similar connection to the topic of Reading Comprehension skills. Hu and Yu [18] discovered various aspects of the perceived quality regarding digital reading instruction on teenagers from the PISA 2018 report. Among the variables that stood out were the way the instruction is focused, the way the class is managed, and the supportive atmosphere. There were also positive correlations with the teacher's adaptation of instruction, encouragement of engagement, discipline, and support for and interest in students. Hence, Reading Comprehension skills is studied through its components, yet related research sheds light on this complex phenomenon. Thus, we face a still-expanding field of study.

#### 2.2.1 Playing online games

The relationship between reading comprehension skills and video games is an expanding area of research. Another previous study [43] on the PISA report (2012) with a sample of 145,953 teenagers examined the relationship between video games and reading comprehension skills, taking into account gender differences. For both genders, moderate use of single-player games was associated with an advantage in performance. In contrast, frequent participation in online collaborative games is generally associated with a significant decline in performance, particularly on paper-based tests and among low-achieving students. Therefore, it can be concluded that excessive gaming may hinder academic success, but moderate gaming may improve performance. Interestingly, in several countries, video games explained the difference in the gender gap in reading comprehension skills between paper-based and computer-based assessments.

Other related studies are found on reading comprehension as they addressed the reading process in a more general manner. The findings of Borgonovi concerning video games [43] is even true for digital gamification in the case of children with reading disabilities, who ultimately show improvement [44]. Gromada [45] finds that moderate video game use is positively associated with improved reading in 10-year-old students from six countries. In terms of its impact on lower achievement, intensive gaming is associated with a 26-point drop in scores for low-achieving participants, compared to a 6-point drop for high-achieving participants. Other research supports the idea that the use of video games can improve eye tracking in reading [46], reading performance [47], and reading comprehension [48]. In a neuroscience study, Kress et al. [49] acknowledge that the relationship between attention, gaming, and reading is increasingly being studied. They conclude that gamified experiences positively affect lexical and sublexical aspects of reading attention tasks. Furthermore, peripheral visual demands are associated with improved reading, while they infer that central gaming demands are associated with slower reading.

#### 2.2.2 Daily internet usage (week)

Studies on reading in general have been found, but none specifically on reading comprehension skills. Hartanto et al. [50] studied a sample of 30,000 American adolescents. They concluded that higher weekday video game use is associated with lower performance on standardized assessments of math, reading, and science. Conversely, weekend video game use is positively associated with academic achievement. They suggested that these findings may be due to the context in which they occurred. Hilt [51] found that Internet addiction is negatively related to deep learning, attitudes toward reading, and reading habits. In contrast, excessive internet use is positively correlated with shallow learning.

#### 2.2.3 Perceptions of information and communications technology and autonomy

Peng et al. [19], through statistical analysis of AI algorithms and hierarchical linear models, relate ICT literacy, autonomy, preference for different reading formats, and schools with technological resources or technology promotion policies to higher reading comprehension skills in the 2018 PISA report.

A more general study on reading has been found. Sarimanah et al. [52] examine the following factors: students' economic capability, attitudes towards ICT, and reading achievement. They conclude that attitudes towards ICT have a significant effect on reading achievement, and the direct effect of students' economic ability on attitudes towards ICT was positively significant. Attitudes toward ICT positively mediate the relationship between students’ economic literacy and reading achievement for the mediation analysis.

#### 2.2.4 Parental education level

Studies on reading and achievement have been found to generally impact reading comprehension skills. The relationship between parental influence and reading comprehension skills is a less explored area. Eiberg and Olsen [53] suggest that the educational expectations of adoptive mothers’ influence reading achievement. Cutler [54], in an observational study of shared reading between parents and children, shows that mothers engage in more physical behaviors while fathers engage in more connected physical contact with their children.

Specific studies on reading comprehension concerning skills have been found. Novita et al. [55] found a correlation between parents' and teachers’ judgments of students' math and reading skills and students' achievement in these skills. In an earlier educational stage, the early childhood stage, various studies discuss the influence of a higher educational level of the mother on the better reading skills of the children [56]. In addition, it is noted that the reasons that motivate mothers are developing cognitive skills, such as expansion of knowledge, vocabulary, reasoning and imagination, in children [57]. Other studies underline developing emotional aspects on children [58].

### 2.3 Reading comprehension skills in Spain: their current situation

One of the main references for the acquisition of reading comprehension skills (in addition to mathematics and science) by young people in different countries is the PISA report. The most recent one was conducted in 2018 and revealed a number of noteworthy aspects in which we can relate the use of technology in social networks or online games by teenagers and their academic performance [20]. The report showed that excessive use of technology among 15-year-olds can have a negative impact on their performance. Students who spent more time in front of electronic screens, especially on weekdays, tended to perform worse on PISA tests [5]. Gender differences in technology use were also observed. Boys tended to be more prone to activities such as online gaming, which was often associated with a more negative impact on their grades. On the other hand, girls showed a better balance between technology use and studying [39].

In the 2018 PISA report, Spain performed slightly below the OECD average in reading comprehension skills and mathematics. The variability in educational performance between regions in Spain is also notable [59]. This highlights the need to address inequalities in the education system and ensure that all students have access to quality education, regardless of their geographical location. Education policies are needed to address the shortcomings identified in the report, such as strengthening mathematics education and promoting reading comprehension skills.

Several articles have used machine learning techniques to analyze the 2018 PISA report. For example, Tan & Cutumisu [60] examined self-efficacy and its impact on learning experiences and overall well-being. Their findings showed that non-cognitive factors, such as a sense of purpose in life and motivation, significantly influenced students. In addition, students who were highly motivated also reported better psychological well-being [61]. Meanwhile, Hu & Yu [18] conducted a comparative study spanning PISA reports from 2009 to 2018 and found that the positive use of social networks for recreational purposes can improve students’ reading comprehension.

In the specific context of PISA 2018 in Spain, Navarro-Martinez & Peña-Acuña [11] examine the impact of recent technological advances on society and their significant influence on education. This study examines the relationship between students’ academic performance (including Sciences, Reading comprehension skills and Mathematics) and their use of technology and social networks. The results suggest that excessive use of technology and social networks has a negative impact on academic performance. Although the previous work by Navarro-Martinez & Peña-Acuña [11] presents valuable research on the relationship between electronic device use and academic outcomes, it did not take into account several critical variables that could potentially influence the results. Including these additional variables would provide a more comprehensive perspective on the phenomenon. For example, socioeconomic factors, school type (public or private), geographic location (Spanish regions), or parental education level could be considered.

### 2.4 Use of machine learning models with explainability in the context of data analysis

It is important to note that within the field of artificial intelligence (AI), there are specialized machine learning (ML) algorithms designed to detect patterns in data and make predictions autonomously, a process known as supervised learning. These systems typically involve two key steps: first, they use algorithms to train models on available data, and second, these trained models are used to make predictions on unfamiliar data. ML is increasingly finding applications in various domains, including industry [[62], [63], [64]], healthcare [65,66], and especially education [67,68].

Historically, a significant barrier to the widespread adoption of ML algorithms in data analysis has been the challenge of interpreting their results, often colloquially referred to as “black boxes” [69]. ML algorithms include different learning styles, such as neighborhood-based algorithms, decision trees, support vector machines, or neural networks. These algorithms are capable of discovering relationships between variables, both linear and nonlinear. This versatility makes the resulting models more suitable for capturing complex data distributions than purely linear models, as explained in the study by Liang et al. [70]. It is common practice to experiment with different algorithms for a given problem and select the one or ones that provide the most favorable results for making predictions.

In addition, recent advances in explicable AI (XAI) [21] have made it more accessible to extract valuable and understandable insights from machine learning (ML) models (XAI-ML). In particular, post-hoc techniques provide a unified approach to explain the predictions generated by ML models. An example of such a tool is SHAP (SHapley Additive exPlanations), developed by Lundberg [71], which relies on game theory principles and allows us to identify the importance of variables at a general, group, or individual level.

## 3 Methods

### 3.1 Participants and context

The PISA report is a global assessment conducted every three years under the auspices of the Organization for Economic Cooperation and Development [20]. Its main objective is to measure and compare the academic performance of 15-year-old students in different countries by analyzing their performance in mathematics, science and reading. It provides a perspective on the quality of education in different nations, identifying strengths and weaknesses in their education systems. It serves as a fundamental tool for understanding and improving education worldwide. It provides detailed information about students’ skills and competencies, helping to identify areas for improvement and to make informed decisions to implement more effective education policies.

The PISA study is regarded as a quasi-experimental research design, given that students are selected by schools. While the study's designers strive to control and balance the samples as much as possible, the allocation is non-random, precluding its classification as an experimental design. Consequently, the study design possesses certain limitations in terms of establishing definitive causality. Nonetheless, it still furnishes valuable comparative insights into educational performance across international contexts.

Microdata of PISA2018^1^ Spain contains about 36,000 samples of student background variables, schools, and academic test scores, of which 22,400 (67 %) contain all the information needed to conduct our study. It includes all students aged between 15 years and three full months and 16 years and two full months at the start of the assessment period and allows countries to vary these cut-offs by up to one month.

A summary of the data is shown in Table 1, the sampled number of students is similar in terms of gender in most regions, and the most numerous are Madrid, Galicia, Murcia, Asturias, and Andalusia. As for the best regions with the highest average rates in the reading tests are Castile and Leon, Asturias, Murcia and Galicia, their average being above 500 points.

**Table 1:** Number of samples in the data studied by region of Spain (N), type of school and gender of student. The values of M and SD are the mean and standard deviation of the reading. The highest values in reading are marked in bold.

Region	N	N - Private		N - Public		Reading	
		Female	Male	Female	Male	M	SD
Madrid	3106	815	825	729	737	497.7	86.8
Basque Country	1656	504	495	323	334	483.1	88.3
Asturias	1427	235	192	503	497	**510.8**	82.4
Galicia	1415	202	199	506	508	**507.1**	81.6
Cantabria	1380	232	204	485	459	497.0	79.3
Castile and Leon	1373	252	239	447	435	**512.0**	81.2
Murcia	1187	185	189	403	410	**507.8**	85.1
Navarre	1177	272	289	302	314	494.8	85.0
Andalusia	1174	120	134	467	453	483.9	84.0
Castile-La Mancha	1125	111	113	434	467	495.0	83.1
Aragon	1094	213	210	313	358	499.4	87.0
La Rioja	1047	239	237	305	266	481.6	83.8
Catalonia	1024	183	186	329	326	494.1	88.5
Valencian Community	1017	197	185	315	320	488.3	83.6
Extremadura	973	126	105	379	363	480.0	80.8
Canary Islands	959	136	142	330	351	487.6	82.1
Balearic Islands	924	141	138	299	346	489.2	83.0
Ceuta	204	69	53	36	46	440.6	83.7
Melilla	154	21	21	48	64	466.6	86.0

### 3.2 Data description and focal variables

The focal variables are the predictors of the present study. They are part of the variables collected in the different self-report forms of students, schools, and their scores in different competencies of the PISA 2018 test. We have classified them into different dimensions such as home, technological, ability perception, geographic, scholar and other factors, as well as performance in the reading test. Table 2 lists the selected variables and their dimensions. With regard to the educational scales, which are noted in ISCED (International Standard Classification of Education) levels maintained by UNESCO (United Nations Educational Scientific and Cultural Organization), and the scale represents 0 to 3 levels for teenagers corresponding to: <1) no education; 1) primary education; 2) lower secondary education; 3B–C) vocational/pre-vocational upper secondary education; 3A) general upper secondary education.

**Table 2:** Description of the variables used in this study.

Dimension	PISA code	Description	Type	Values
Home	ST005Q01TA	Mother's level of education	Categorical	[<1, 1, 2, 3B–C, 3A]
	ST007Q01TA	Father's level of education	Categorical	[<1, 1, 2, 3B–C, 3A]
	PA042Q01TA	Annual household income	(Excluded)	Not completed in Spain
Technological	IC002Q01HA	Age at first use of digital device	Categorical	[<3 years, 4–6, 7–9, 10–12, >13]
	IC006Q01TA	Daily Internet usage (week)	Categorical	[No time, 1–30', 31'-60', 1–2, 2–4, 4–6, >6]
	IC007Q01TA	Daily Internet usage (weekend)	Categorical	[No time, 1–30', 31'-60', 1–2, 2–4, 4–6, >6]
	IC008Q05TA	Social networks participation	Categorical	[No time, 1–30', 31'-60', 1–2, 2–4, 4–6, >6]
	IC008Q07NA	Playing online games	Categorical	[No time, 1–30', 31'-60', 1–2, 2–4, 4–6, >6]
	IC013Q05NA	Usefulness of social networks	Categorical	[Strongly disagree, Disagree, Agree, Strongly agree]
Skill perception	COMPICT	Perceived ICT competence (WLE)	Numeric	[-3.0: +3.0]
	AUTICT	Perceived autonomy related to ICT use (WLE)	Numeric	[-3.0: +3.0]
Geographic	Region	Regions of Spain	Categorical	[Andalusia, Aragon, Asturias, Balearic Islands, Canary Islands, Cantabria, Castile and Leon, Castile-La Mancha, Catalonia, Extremadura, Galicia, La Rioja, Madrid, Murcia, Navarre, Basque Country, Comunidad Valenciana, Ceuta, Melilla]
Scholar	SC013Q01TA	Type of school	Categorical	[Public, Private]
Gender considerations	ST004D01T	Gender	Categorical	[Male, Female]
Reading comprehension skills	PVREAD	Mean of reading test per student	Numeric	[0 : 1000]

WLE stands for Weighted Likelihood Estimates.

The reliability of students’ self-reported scale scores is available in the PISA technical reports.

### 3.3 Data analysis

For our analysis, we took an ML approach by testing and validating several algorithms. We chose a specific metric that is commonly used in this type of study to evaluate the results.

#### 3.3.1 Machine learning approach

Recent approaches to data analysis using ML models on PISA report data can be observed in studies such as [60], where the authors test a couple of ML algorithms to conduct the study and make comparisons, and Miao et al. [72], where the authors employ a single algorithm for the entire study. In our research, aiming to enhance the robustness of our results, we will assess multiple machine learning algorithms representing diverse learning styles. This approach enables us to select the algorithm that best predicts outcomes, providing more accurate and interpretable results. These selected styles can be categorized as neighborhood-based, decision trees, Bayesian models, and neural networks.

Below is a brief description of each of the selected algorithms:

● Baseline: This serves as our reference model, where all predictions are assumed to be equal to the mean of the training set.
● Linear Regression: This regressor calculates multiple linear coefficients of the predictor variables and an intercept value using a least squares approach. Variants such as Ridge [73], LASSO [74], and ARD [75] have also been used, where the optimization objective may vary.
● Decision Tree [76]: This model uses a hierarchical structure constructed from a dataset by selecting the most relevant variable and applying a cutoff value to create two subsets of samples. This process continues recursively until a stopping criterion is met.
● Random Forest [77]: This algorithm constructs multiple decision trees and combines their predictions, resulting in more robust behavior.
● AdaBoost (Adaptive Boosting) [78]: AdaBoost uses several linear regressors, and the final prediction is determined by weighting the regressors according to the confidence learned during the training phase.
● Gradient Boosting Trees: These algorithms use boosting techniques combined with the creation of multiple decision trees. They optimize using derivable cost functions and update weights with gradient descent, similar to neural networks. There are different implementations of each algorithm that use different optimizers and have shown strong performance in open challenges. The ones we used are eXtreme Gradient Boost [XGBoost] [79], an algorithm that transforms numerical values into categorical features [CatBoost] [80], Light Gradient Boosting Machine [LightGBM] [81], and Histogram-based Gradient Boosting Regression Tree [Histogram GBR] [82].
● Multilayer Perceptron [MLP] [83]: This basic neural network is widely used and has connections between all neurons in successive layers.
● Nearest Neighbors [NN] [84]: Calculates the class of a new sample based on the k (parameter) nearest training samples and their associated classes. The Euclidean distance is commonly used as the nearest neighbor function, and the parameter k is set to 1, 3, 5, and 7 in our study.

To evaluate the performance of predictive models, we will use the widely accepted 10-fold cross-validation method. To compare different models, we will use Root Mean Squared Error (RMSE), a common metric in regression analysis. RMSE quantifies prediction accuracy by calculating the square root of the sum of squared errors (differences between actual and predicted values) divided by the number of samples. Notably, RMSE penalizes large prediction errors and is expressed in the same units as the original measure, making it easier to interpret.

#### 3.3.2 Preprocessing data

In our case, the numerical variables are already scaled using weighted likelihood estimation [85], so no transformation is necessary, but with respect to the categorical variables, the one-hot technique was used, which consists of replacing each variable that is represented as a column in the data table with as many additional columns as there are categories. For each sample, these additional variables would have a value of 0 and only the column corresponding to the true category of the sample would have a value of 1.

By changing the representation of the original data to these common transformations, we facilitate the learning of certain types of algorithms, such as neighborhood-based or linear algorithms.

#### 3.3.3 Machine learning and explainability

The most accurate predictions often come from complex machine learning models. To understand why these models consistently make accurate predictions, a post hoc approach can be applied after the model is trained.

**Fig. 1:**
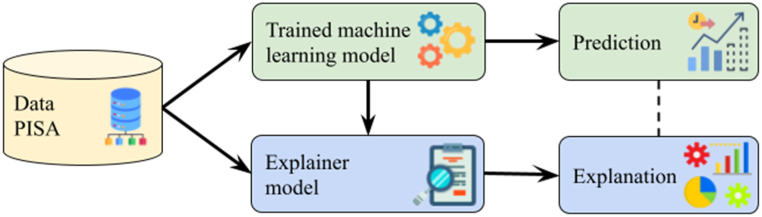
General scheme of the post-hoc explicability of machine learning models.

These issues can be approached in two ways. The first method involves controlled permutations of independent variables to assess their impact on the dependent variable, thereby estimating variable importance [77]. The second method (Fig. 1) uses game theory and Shapley values [86], which determine the marginal contribution of each independent variable considering all possible combinations with other variables to explain the final outcome. This method ensures local accuracy, consistency and missingness. This information is then used to provide feedback on the prediction process.

Shapley values come in handy when we need to understand the overall impact of a variable but struggle to explain its influence on a group of samples or a specific sample. Lundberg and Lee [87,88] have made significant progress in addressing these challenges. One valuable tool that follows this approach is SHAP (SHapley Additive exPlanations) [71], which enables explainability at the global, group, or individual level.

## 4 Results

### 4.1 Selection of the machine learning model

To address the research questions, we must first select a machine learning model and then apply explainability techniques to it once it is trained.

**Fig. 2:**
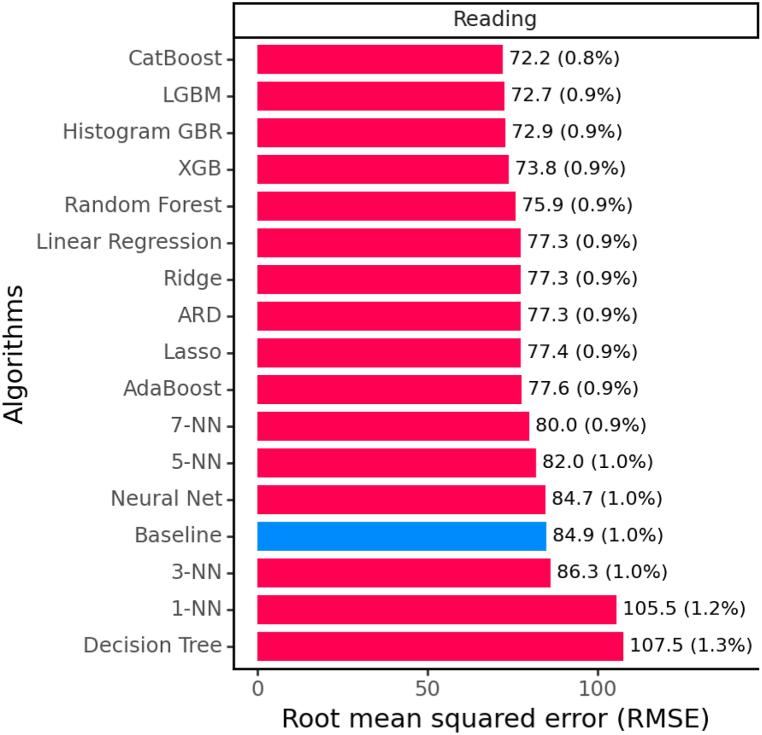
Average metric results of 10-fold cross-validation. The number in parentheses represents the improvement ratio with respect to the baseline (current result/baseline result). The higher value is better. Baseline is marked in blue. (For interpretation of the references to colour in this figure legend, the reader is referred to the Web version of this article.)

Fig. 2 shows the average results of the cross-validation using RMSE.^2^ It is also important to compare the results in order to determine whether the differences between the algorithms are statistically significant. To achieve this, we employ the Wilcoxon paired test [89] with a 95 % confidence level, which is a commonly accepted threshold.

**Fig. 3:**
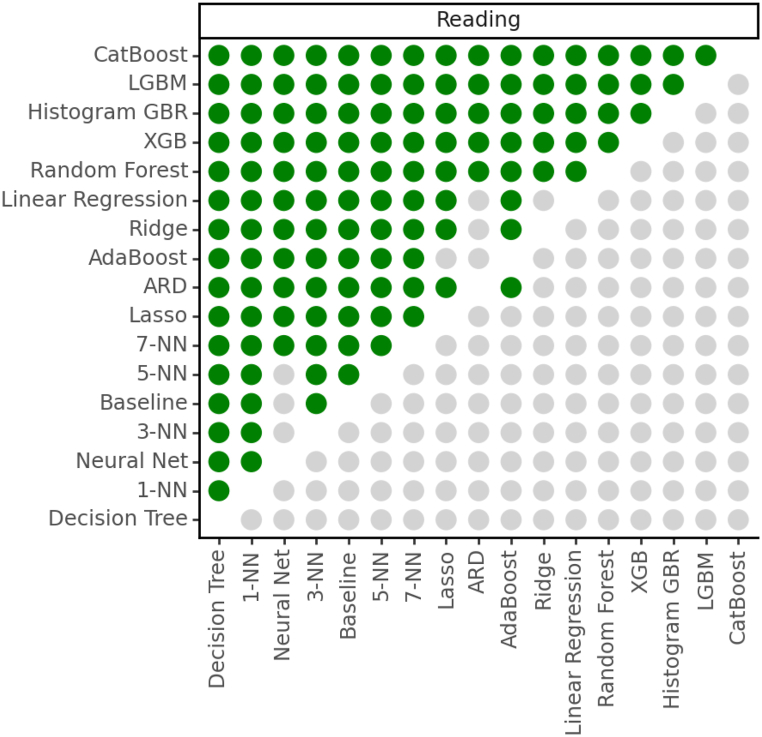
Results of the Wilcoxon 10-fold cross-validation test according to the RMSE values. Green bullets indicate that the row algorithm is significantly better than the column algorithm at 95 % confidence. (For interpretation of the references to colour in this figure legend, the reader is referred to the Web version of this article.)

Fig. 3 shows a comparison of the statistical significance between pairs of algorithms according to the RMSE error metric. It can be seen that: i) Baseline, Multi-Layer Perceptron, Decision Tree and K-nearest neighbors perform the worst; ii) The best algorithms are based on gradient boosting algorithms on multiple decision trees (CatBoost, LGBM, Histogram GBR, XGB and Random Forest); CatBoost is significantly better than the rest, so we will select it to address the explainability section.

In addition, we also performed preliminary tests on some partitions with hyperparameter optimization algorithms [90,91] on models based on gradient boosting, because despite the high computational cost with respect to learning with the default parameters, sometimes this parameter adjustment is justified by the improvement in the results. In this case, the differences obtained were not better with respect to the default parameters, so we ruled out performing the full tests with cross-validation.

### 4.2 The relevance of factors involved in this study in explaining reading comprehension skills in Spanish teenagers

The importance of the variables is based on their ability to explain the target variable, Reading comprehension skills. In our case, we use the CatBoost algorithm based on the results obtained in the previous subsection.

**Fig. 4:**
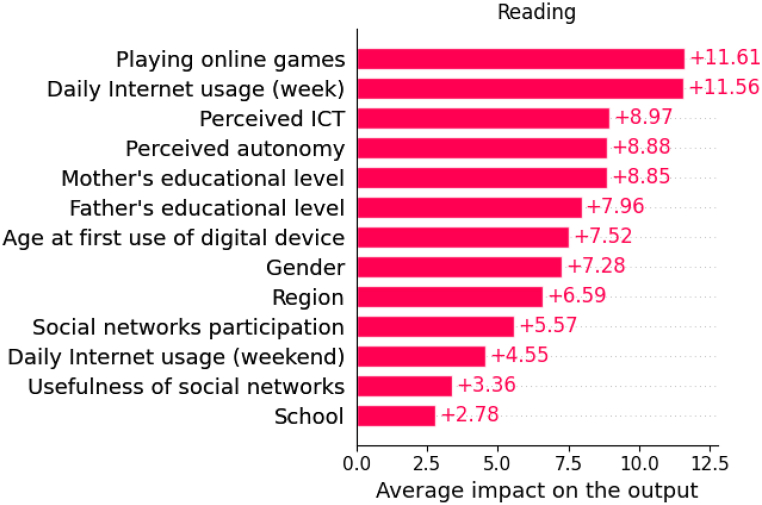
Average importance of predictors and their impact on the outcome variable (Reading).

In this trained model, Shapley values are calculated for each variable and their cumulative absolute values determine their importance. Fig. 4 shows the model's predictors in order of importance. In what follows, we present the estimated impacts as calculated by the model using SHAP values, and we break them down by sample, focusing on the most relevant variables. It is important to keep in mind that theoretical PISA scores range from 0 to 1000. In practice, the best performing region in Spain has an average reading comprehension skills score of 510.8 (Asturias), while the worst performing region has an average score of 480.0 (Extremadura) (see Table 1), which is a remarkable difference of 30.8 points. This difference serves as a gap for comparing the impacts shown in Fig. 4 and the following figures, which provide estimates of the impact values for each of the variables studied. It is worth remembering that the approximate difference between an academic year is around 30 points [39].

The variable with the highest average impact is *Playing Online Games*, which is 11.61. This value represents slightly more than half of the 20.8-point difference observed between the top and bottom performing Spanish regions and corresponds to about one third of the difference in an academic course level.

The use of the Internet also appears to be very relevant. This is followed by participants’ perceptions of their digital literacy and autonomy. They show lower values than the previous variables, but also have an impact of around 9 points on reading achievement. Other variables with an average impact of between 8 and 9 points are the educational level of the parents. The results are also very similar for both parents.

The next variables in terms of their impact are the age at which they started using digital devices and the gender of the participants. Finally, we observe the rest of the variables that, although they have an impact on our model, their average impact is less than 7. These variables are the region of the participants, the participation in social networks, the daily Internet use on the weekend, the usefulness of social networks and the type of school, public or private.

### 4.3 What is the relationship between the values of the most important factors with respect to reading comprehension skills, and the advisable recommendations

To explain in detail the relationships between the variables studied and reading comprehension skills (in tables this variable appears with the term *Reading*), we will focus on the most important ones, as shown in Fig. 4. In the following, we will present some of the figures in pairs since they share certain common concepts.

**Fig. 5:**
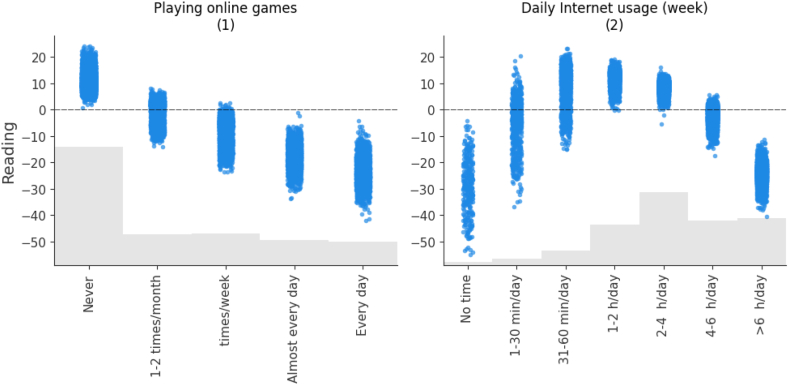
The effects of the model on the technology adoption variables. Each subgraph shows the name of the variable, with the order of importance between them in parentheses.

Fig. 5 shows the individual impact scores for technology use. Playing online games shows a negative effect as its frequency increases. Students who never play online games score up to 20 points higher, while the majority of those who play everyday score between 15 and 40 points lower. Much lower values are especially noticeable when these games are used practically every day. On the contrary, the daily use of the Internet shows a bell shape, that is, a positive effect in the central ranges that include from 1 h/day to 4 h/day, being negative at the extremes, both when it is not used and when it is used more than 4 h/day. For both variables, the average impact value is above 11 points, namely 11.61 for playing online games and 11.56 for daily internet use.

**Fig. 6:**
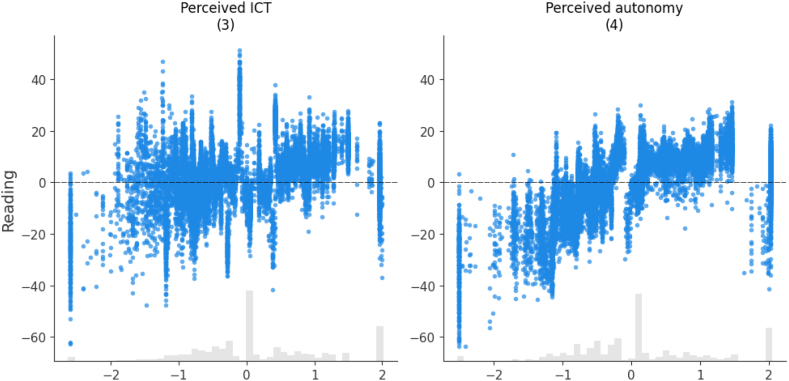
Perceptions of autonomy and technology literacy by teenagers. Each subgraph shows the name of the variable, with the order of importance between in parentheses.

Fig. 6 shows a strong and direct relationship between two key factors: perceptions of autonomy and technology literacy and their impact on teenagers’ reading comprehension. As we can see, better perceptions of autonomy or technology literacy are associated with better reading comprehension skills. In both cases, there is a similar upward trend, although it is visually more obvious for perceived autonomy. In the latter case, students score significantly higher than those who perceive less autonomy, with a difference of up to 50 points. Similarly, a positive perception also translates into better reading comprehension skills, with improvements of up to 30 points. The average impact score is very close to 9 points in both cases. It is 8.97 for perceived ICT and 8.88 for perceived autonomy.

**Fig. 7:**
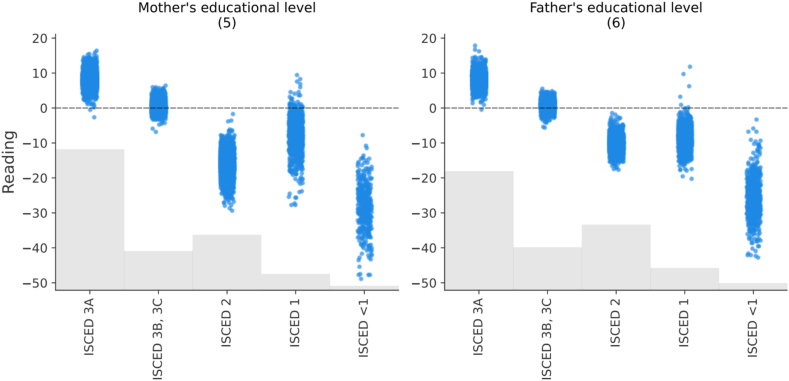
The effects of the model on the level of studies completed by parents. Each subgraph shows the name of the variable, with the order of importance between in parentheses.

Fig. 7 highlights an apparent decreasing relationship between parents' educational attainment and reading comprehension skills. When parents have completed at least secondary education (3A), teenagers' scores improve by an additional 0 to 15 points. As the parents' level of education decreases, so do the scores, in some cases reaching a gap of more than 40 points when the parents have not even completed primary school. This behavior is replicated for both fathers' and mothers' education, underscoring the crucial influence of parents' education on their children's academic performance. The average impact in the case of the mother's educational level is 8.85 and the father's 7.96.

**Fig. 8:**
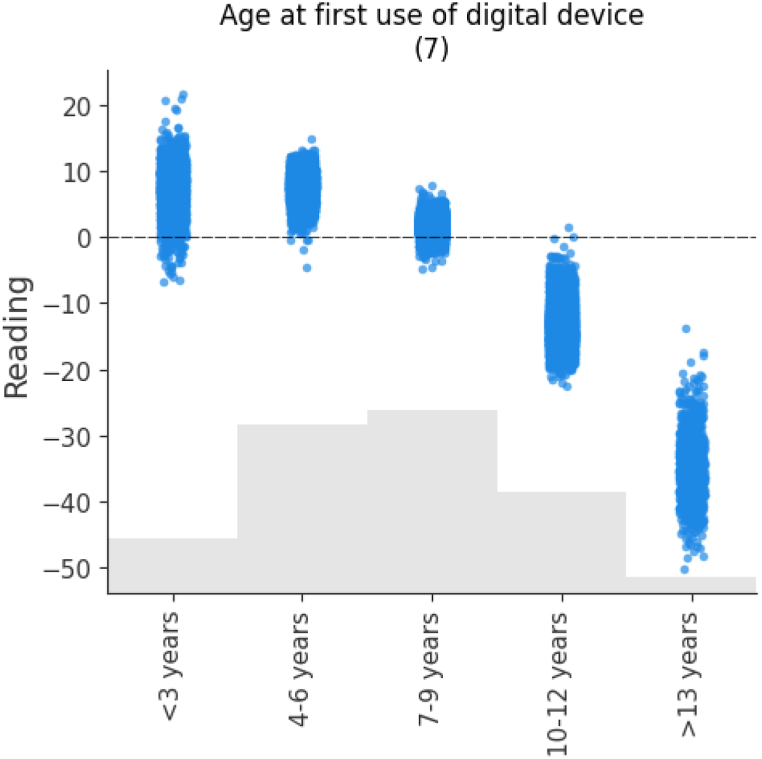
The effects of the *Age at First use of digital Device*.

Fig. 8 shows that there is an inversely proportional relationship between the age at which digital devices were first used and reading comprehension skills. Higher scores are obtained when starting at a very young age, and scores decline with increasing age. In most cases, a higher score is obtained if the first use of devices occurs before the age of 10, up to 15 points if it occurs in pre-primary school. On the other hand, if this first contact takes place between the ages of 10 and 12, the score is up to 20 points lower, and even between 20 and 50 points lower if they use devices for the first time from the age of 13 onwards.

**Fig. 9:**
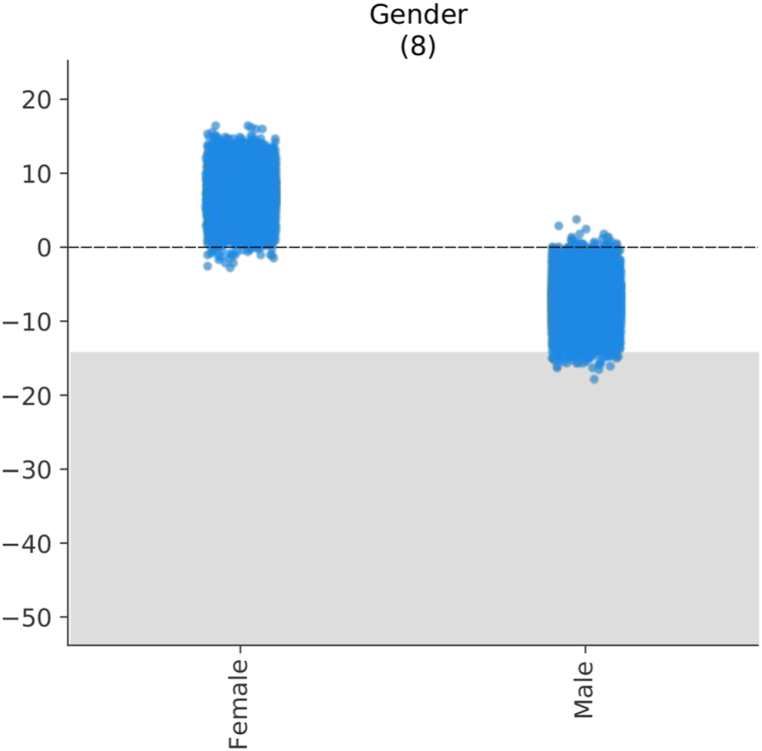
The impacts of *Gender*.

Regarding Fig. 9, the data show a difference in the development of reading comprehension between male and female teenagers. Girls tend to score higher, with an average advantage of about 0–15 points. For boys, on the other hand, the opposite is true in the same proportion, up to 15 points lower. The average impact score is still 7.28.

The advisable recommendations would be related to the values of the variables studied. As a compendium of positive impact scenarios derived from the detailed impact graphs (Fig.s 5 through 9), we present in Table 3 a series of statistically contrasted recommendations using the nonparametric Mann & Whitney *U* test (1947). This test compares the reading test scores of two groups, focusing on only one variable. The first group consists of students who meet the specified criteria, while the second group consists of those who do not. As shown in the table, students who meet the criteria show significantly higher performance levels, with a confidence level of more than 99 %. It is noteworthy that in the case of the combination of all the criteria (bottom row, representing 2 % of the total), the mean score reaches 555.5, which is higher than the values observed for any of the previous criteria and, in particular, higher than the average performance for the case of Spain.

**Table 3:** Criteria with positive impact on reading by variable contrasted by Mann-Whitney *U* test. N (%) represents the percentage of students meeting the criterion relative to the total number of students, and M is the mean.

Variable	Satisfy the criterion			Do not satisfy the criterion	U	p-value
	Description	N (%)	M	M		
Playing games	No play	52	510.3	478.4	27.8	<0.001
Daily Internet usage (week)	From 1h/day to 4 h/day	49	508.8	481.2	24.5	<0.001
Perceived ICT	Positive value	61	498.3	489.5	6.9	<0.001
Perceived autonomy	Positive value	55	498.3	490.7	6.1	<0.001
Mother's educational level	Maximum level (3A)	55	510.0	476.3	29.2	<0.001
Father's educational level	Maximum level (3A)	46	511.6	480.3	27.8	<0.001
Age at first use of digital device	Less than 9 years	77	501.6	471.6	22.1	<0.001
Gender	Female	50	506.7	483.0	20.4	<0.001
	All criteria	2	555.5	493.9	13.5	<0.001

## 5 Discussion and conclusion

### 5.1 Relevant factors related to comprehension reading skills in Spanish teenagers

This investigation provides significant results that represent a holistic deepening of the reading comprehension skills, a specific field that is expanding. We will present studies directly related to reading comprehension skills to enrich the discussion and understand the scope of these findings, alongside other studies that share a similar connection to the broader phenomena of reading comprehension or the reading phenomenon.

Primarily, the first contribution of this research is that the research provides an inventory of interrelated impacts of eight most important variables. According to the results, the following factors stand out in descending order of importance for the reading comprehension skills of Spanish teenagers: 1) *Playing Online Games*; 2) *Daily Internet Usage* (week); 3) *Perceived ICT*; 4) *Perceived autonomy*; 5) *Mother's educational level*; 6) *Father's educational level*; 7) *Age at First use of digital Device*; and 8) *Gender*.

Secondly, *Playing Online Games* is found to be the most important variable explaining the negative impact on Reading Comprehension skills. Thirdly, the variables that emerge for the first time that allow explaining reading comprehension are *Perceived ICT* and *Parental Education Level*.

Fourthly, *Daily Internet Usage (week)* and *Age at First Use of Digital Device* provide a broader perspective than a linear correlation on how ICT usage influences reading comprehension skills compared to the study by Navarro-Martínez & Peña-Acuña [11]. These new nuances provided allow for a more detailed understanding of how these variables interrelate with each other. Navarro-Martínez and Peña-Acuña [11] studying the frequency of ICT and social media usage, suggest that early and frequent use of digital devices and the internet is associated with lower academic performance, concerning Reading comprehension skills, Sciences and Mathematics. They note a negative relationship between early adoption of the internet and digital devices and results in PISA tests 2018. In contrast, this study focusing on Reading Comprehension skills refines the use of the internet within the 1–4 h range as beneficial, not referring in this exploration to the variable in general but to the influence of its different categories. All these findings answer RQ2.

The quality of this study lies in the investigation of three cognitive skills: literate, inferential, and evaluation, similar to the study conducted by Huh [14,15] and other studies related to the PISA report [18,19,43,50]. However, this study differs from other studies because it addresses different age groups or in relation to other variables. However Huh [14,15] which focuses on the same variables, caters to a narrower age range of students. Other studies of comprehension skills interconnect this variable with other abilities or aspects. Landi [12] explores it in relation to spelling, while Kang et al. [13] investigate its connection with reading fluency. Hu and Yu [18] focuses on quality perception regarding digital reading instruction.

Regarding the use of online games, it is consistently negative, with a gradual decline in performance as the time spent with these devices increases. This finding is consistent with the study from the PISA 2012 report by Borgonovi [43] and Hartanto et al. [50] on reading comprehension skills. However, moderate, and appropriate use of the Internet can be beneficial for students of this age, as suggested by previous study on reading comprehension skills of Borgonovi [43]. It also aligns with studies on reading [16,45]. An interesting finding in this research is that daily use for more than 4 h is as detrimental as no use at all. Hence, according to the results of this research and considering the summary of recommendations (Table 3), the use of technology can have a positive impact on the reading habits of Spanish teenagers [27].

If applicable, Hilt [51] clarifies that in the case of addiction, there is a positive correlation with shallow learning and a negative correlation with attitude, reading habit, and deep learning. The ideal time of use is more than 1 h but does not exceed excessive use of more than 4 h. These results add nuances to the previous study that dealt with similar variables and reached analogous conclusions [11].

Based on the findings of this study, it is concluded that moderate use of ICT, focused on learning and with established time limits, is advisable. Although online games can be entertaining, their abuse can lead to a decrease in academic performance and the acquisition of important skills such as reading Lareki et al. [32]. Therefore, teenagers and their families should set limits and guidelines for the amount of time spent playing these games. At the same time, there is a need to take advantage of the educational benefits of Internet-mediated learning, as well as attitudes toward ICT and reading [52]. Not all aspects of technology are harmful, and it can be a valuable tool for learning when used appropriately [24].

The relationship between perceptions of autonomy and reading achievement underscores the importance of empowering teenagers in their educational process, consistent with the findings of Peng et al. [19]. When students feel autonomous, they have a greater sense of control over their learning, which leads to increased motivation and engagement in reading [35] in a self-regulated learning situation [92] and perceived self-efficacy [93]. This relationship suggests that it is not just about teaching reading skills, but also about fostering a supportive climate [94] that promotes students’ confidence and autonomy. Indeed, as García et al. [25] argue, the better adolescents perceive this, the higher their educational achievement will be.

In terms of the direct relationship between technology use skills and reading comprehension performance, consistent with Peng et al. [19], it underscores the growing importance of incorporating digital literacy into contemporary education. Technology has become an integral tool in everyday life and academia, enhancing reading comprehension by providing access to additional information and facilitating more interactive learning practices [95].

A novel contribution of this study is to discover the extent of the influence of mothers with higher levels of education on teenagers’ greater reading comprehension ability. This is a novel contribution of this research as this segment has not been studied as extensively. A study in the early childhood stage is consistent with the claim that the influence of mothers with higher educational levels predictively contributes to the greater reading ability of children [56].

This study suggests that the educational environment [19] and parents' educational level have a significant impact on teenagers' reading comprehension skills. In addition to these factors, a higher socioeconomic level, which is often associated with parents' educational level and their support of their children's learning, can influence achievement and literacy [93]. Active supervision and adult support play a crucial role in the development of effective learning habits [5]. In this regard, Gavora [57] conducted a study among mothers with high and low levels of education and concluded that mothers with higher levels excelled in developing cognitive aspects and valued reading time with their children. While mothers with lower levels outperform the others in explaining to children why they should read.

In line with the above, an early introduction to digital devices in preschool education can foster interest in reading comprehension and improve cognitive skills. A late start, from the age of 9, may hinder adaptation to digital environments and affect reading literacy [96]. Finally, the gender of teenagers determines differences in reading comprehension skills in favor of females.

Although this study shows that there may be discrepancies in the results based on other variables used, their results were not considered due to their limited influence. Some differences were found between autonomous communities, but they were less significant than the variables mentioned above. The use of social networks was not relevant in this research, although other studies have linked excessive use of these networks to performance in mathematics, science, and reading [97]. Similarly, the use of the Internet on weekends or the type of educational institution, public or private, was not significant in this study.

In conclusion, in the present study, we used regression-based machine learning models to investigate the relationship between various factors related to home environment, technology, perceived ability, geographic location, school-related aspects, gender, and academic performance in reading comprehension skills among Spanish teenagers. First, a series of models corresponding to different learning styles were used to identify the most appropriate one. Subsequently, the application of explanatory techniques allowed us to evaluate the impact of these variables, highlighting the most salient factors. Specifically, we observed that moderate Internet use, non-participation in online games, positive perceptions of ability, higher parental education level, early exposure to technology, and being female are positively associated with academic performance. These findings underscore the importance of a holistic approach to education that encompasses autonomy, technological literacy, and addresses socioeconomic and gender disparities in education. All these findings answer RQ1.

### 5.2 Theoretical contributions and implications

Notably, the findings reveal an adverse correlation between online gaming and reading comprehension skills, particularly with extended exposure time. It is imperative to recognize that the current landscape of online gaming does not promote reading comprehension skills. On the other hand, moderate daily Internet use (between 1 and 4 h per week) was identified as a positive factor. This suggests that when individuals engage with the Internet within a balanced time frame, typically between one and 4 h per day, they tend to exhibit improved reading comprehension skills. This underscores the critical role of careful technology selection in educational settings to effectively promote specific skills.

The acquisition of reading comprehension skills cannot be fully addressed unless students have a strong sense of autonomy and technological literacy. Creating an educational environment that fosters these dimensions can have a positive impact on both academic and personal outcomes for teenagers. In addition, this study underscores the importance of introducing technology in a balanced and supervised manner from an early age. This underscores the temporal dimension of the discussion, emphasizing that laying the foundation for literacy and reading comprehension skills literacy begins in childhood and requires ongoing attention and guidance.

This study thus emphasizes the need for comprehensive educational approaches that address both psychological aspects and practical skills. The strategic use of technology in education should not only facilitate the acquisition of academic knowledge, but also the development of critical skills for the modern world, including autonomy, digital literacy, and ensuring equal access to educational opportunities. Such an approach is critical to improving overall educational outcomes and cognitive skills development.

The central role of parental education level is evident in the significant improvement in teenagers' academic performance. Teenagers with educated parents who have attained higher levels of education tend to show substantial improvements. Consequently, this discovery emphasizes the positive influence of nurturing home environments, where parents can offer valuable support and resources that enhance their children's literacy development, thereby improving their reading comprehension skills. Therefore, educational policies and interventions should take into account the crucial role of parents in promoting literacy.

Regarding the first interaction with electronic devices, it can be concluded that a late introduction, starting from the age of 9, has negative associations with their reading comprehension skills. Therefore, we recommend an early and balanced introduction to promote the effective use of resources and facilitate the improvement of their reading comprehension skills.

Effective education requires addressing socioeconomic and gender disparities, which should be addressed at the educational and legislative levels. Ensuring that all students, regardless of their socioeconomic background, have access to quality educational resources and equitable opportunities is essential. In addition, the central role of parental involvement and educational attainment underscores the importance of fostering collaboration between schools and families. These findings are consistent with the need for an inclusive and equitable education model and provide a basis for practical legislative applications. As such, we should promote awareness campaigns, especially targeting parents with lower educational backgrounds.

### 5.3 Limitations and future directions

Despite the interesting results obtained in this research, there are certain limitations. The selection of variables from among the more than 1,000 provided by the PISA report was initially challenging and was done considering the Spanish context. It is also important to consider that there may be cultural and contextual differences between the different autonomous communities where the data were collected.

Caution should therefore be exercised in generalizing the results. However, these limitations also present opportunities for future research that takes these factors into account. Further research could address the impact of social networks on performance or reading comprehension skills, as well as other advanced technologies that are currently in vogue.

At this point, the exposed variables reveal the complexity of the field of reading comprehension skills. In future studies, this research could be compared with other similar contexts to assess their similarities and differences, and to closely evaluate the impact of the cultural contextual factor. Contrasting future PISA studies can show how reading comprehension skills develop in young people. If longitudinal studies with large samples are conducted, it is possible to relate parental education levels to reading performance in primary and secondary school. It would be interesting to conduct more studies on the educational level of the father. This approach could provide consistency in the relationship between the variables studied and different educational circumstances. Definitely, from an applied approach, this research urges comprehensive education strategies and a balanced use of ICT. All these advisable recommendations answer RQ2.

## Funding

This publication is part of the research, development and innovation project (Proyecto I + D + I or R&D Project) Multiliteracies for Adult at-Risk Learners of Additional Languages (MultiLits), REF. PID2020-113460RB-I00, funded by the 10.13039/501100011033Spanish State Research Agency (10.13039/501100004837Ministry of Science and Innovation) 10.13039/501100004837MCIN/AEI/10.13039/501100011033/.

## Data availability statement

The data used for this study are available at http://hdl.handle.net/10045/142844 (Institutional Repository of the University of Alicante) with public and open access. Specifically, the data relating to the *student questionnaire* and the *school questionnaire* in Spain available at https://www.oecd.org/pisa/data/2018database/have been merged.

## CRediT authorship contribution statement

**Juan Ramón Rico-Juan:** Writing – review & editing, Writing – original draft, Visualization, Validation, Supervision, Software, Resources, Project administration, Methodology, Investigation, Formal analysis, Data curation. **Beatriz Peña-Acuña:** Writing – review & editing, Writing – original draft, Supervision, Methodology, Investigation, Formal analysis, Conceptualization. **Oscar Navarro-Martinez:** Writing – review & editing, Writing – original draft, Supervision, Methodology, Investigation, Formal analysis, Conceptualization.

## Declaration of competing interest

The authors declare that they have no known competing financial interests or personal relationships that could have appeared to influence the work reported in this paper.

## Footnotes

1 The microdata from the PISA 2018 report is publicly accessible at https://www.oecd.org/pisa/data/2018database/. We have chosen Spain as the country for our analysis.

2 All these experiments have been carried out with Python (3.10.12) and the libraries scikit-learn (1.2.2), XGBoost (1.7.6), LightGBM (4.0.0) and CatBoost (1.2.1).
